# Revealing the potential role of hsa-miR-663a in modulating the PI3K-Akt signaling pathway via miRNA microarray in spinal muscular atrophy patient fibroblast-derived iPSCs

**DOI:** 10.1093/jnen/nlae065

**Published:** 2024-06-18

**Authors:** Gayatri Gandhi, Radha Kodiappan, Syahril Abdullah, Hoon Koon Teoh, Lihui Tai, Soon Keng Cheong, Wendy Wai Yeng Yeo

**Affiliations:** Perdana University Graduate School of Medicine, Perdana University, Kuala Lumpur, Malaysia; Department of Research and Training, MAHSA Specialist Hospital, Selangor, Malaysia; Medical Genetics Laboratory, Department of Biomedical Sciences, Faculty of Medicine & Health Sciences, Universiti Putra Malaysia, Selangor, Malaysia; Genetics & Regenerative Medicine Research Group, Faculty of Medicine and Health Sciences, Universiti Putra Malaysia, Selangor, Malaysia; Malaysia Genome and Vaccine Institute, National Institutes of Biotechnology Malaysia, Selangor, Malaysia; Centre for Stem Cell Research, M. Kandiah Faculty of Medicine and Health Sciences, Universiti Tunku Abdul Rahman, Selangor, Malaysia; Centre for Stem Cell Research, M. Kandiah Faculty of Medicine and Health Sciences, Universiti Tunku Abdul Rahman, Selangor, Malaysia; Cytopeutics Sdn. Bhd, Selangor, Malaysia; Centre for Stem Cell Research, M. Kandiah Faculty of Medicine and Health Sciences, Universiti Tunku Abdul Rahman, Selangor, Malaysia; Perdana University Graduate School of Medicine, Perdana University, Kuala Lumpur, Malaysia; School of Pharmacy, Monash University Malaysia, Selangor Darul Ehsan, Malaysia

**Keywords:** hsa-miR-663a, microarray, microRNA, PI3K-AKT pathway, spinal muscular atrophy

## Abstract

Spinal muscular atrophy (SMA) is an autosomal recessive neuromuscular disorder due to deletion or mutation of survival motor neuron 1 (*SMN1*) gene. Although survival motor neuron 2 (*SMN2*) gene is still present in SMA patients, the production of full-length survival motor neuron (SMN) protein is insufficient owing to missing or mutated *SMN1*. No current disease-modifying therapies can cure SMA. The aim of this study was to explore microRNA (miRNA)-based therapies that may serve as a potential target for therapeutic intervention in delaying SMA progression or as treatment. The study screened for potentially dysregulated miRNAs in SMA fibroblast-derived iPSCs using miRNA microarray. Results from the miRNA microarray were validated using quantitative reverse transcription polymerase chain reaction. Bioinformatics analysis using various databases was performed to predict the potential putative gene targeted by hsa-miR-663a. The findings showed differential expression of hsa-miR-663a in SMA patients in relation to a healthy control. Bioinformatics analysis identified *GNG7*, *IGF2*, and *TNN* genes that were targeted by hsa-miR-663a to be involved in the PI3K-AKT pathway, which may be associated with disease progression in SMA. Thus, this study suggests the potential role of hsa-miR-663a as therapeutic target for the treatment of SMA patients in the near future.

## Introduction

Spinal muscular atrophy (SMA) is the leading genetic cause of infant mortality and the most frequent autosomal recessive genetic disorder after cystic fibrosis. Although rare, this neuromuscular disease primarily affects children.^1^ It causes progressive proximal muscle weakness and atrophy, which is due to alpha neuron degeneration and irreversible loss in the spinal cord anterior horn.^2^ Mutation or deletion of the survival motor neuron 1 (*SMN1*) gene has been identified as the major contributor to this devastating disease.^3^^,^^4^ The SMN protein, which is present in both neurons and non-neuronal cells is required for normal development and functional homeostasis in almost all species.^5^^,^^6^

There are two *SMN* genes in humans: (i) *SMN1* or telomeric form is crucial for the production of a multifunctional protein, which is known as full-length SMN or FL-SMN, and (ii) *SMN2* centromeric form which is a homologous pseudogene. *SMN2* produces about 90% truncated proteins that rapidly degrade (SMNΔ7) due to the alternative splicing of C  to T transition in exon 7.^3^ The degree of SMA severity is highly variable and the clinical features can be subdivided into five main types (Type 0 to Type IV), from floppy infant to mild adulthood weakness classified based on age of onset, maximum motor function and life expectancy.^7^ Interestingly, the severity of SMA correlates inversely with the SMN protein levels and the *SMN2* copy number.^8^

Recognizing both the individual and societal burden of SMA patients, the need for an effective treatment strategy is essential. At present, three medications were approved by the United States Food and Drug Administration, ie Nusinersen (approved in December 2016), Zolgensma (approved in May 2019), and Evrysdi (approved in August 2020), as treatment options for SMA patients. These therapies are limited due to their high cost, side effects, and invasive route of administration.^9^ Hence, there is a need to explore new biomarkers that are associated with SMA disease processes potential therapeutic targets for future studies that involve follow-up of SMA patients.^10^ This entails a shift in the research focus to the field of epigenetics, particularly miRNAs, that could be a potential therapeutic approach to address the needs of all SMA patients.^11^^,^^12^

MicroRNAs (miRNAs or miRs) belong to a class of small endogenous noncoding single-strand RNAs that are approximately 19-22 nucleotides long and play important roles in post-transcriptional regulation of gene expression.^13^ miRNAs are known to regulate production of protein in the cell cytoplasm by causing either target mRNAs’ degradation and/or inhibiting their translation.^14^ As only partial complementarity is required for a miRNA-mRNA interactions to occur, a single miRNA can potentially regulate thousands of mRNAs.^15^ Changes in miRNA expression could greatly alter the transcriptomic landscape and influence mRNA expression and pathways/processes including those in SMA.^16^^,^^17^ Intriguingly, some studies have reported that miRNAs may induce as well as negatively regulate gene expression.^18^

Emerging reports indicate that aberrant miRNA expression can greatly contribute to pathogenic mechanisms of SMA.^19^^,^^20^ However, the role of miRNAs in SMA type I using induced pluripotent stem cells (iPSCs) model and their target genes remains largely unexplored. Disease modeling with iPSCs are useful for better understanding of diseases and their progression because of their potential to differentiate into any type of cell lineage.^21^ This study focuses on the examination of iPSCs over iPSC-derived neurons in order to gain insights into the broader molecular mechanisms underlying SMA. The selection of using iPSCs cell line allows exploration of molecular dysregulations at a more comprehensive level, encompassing various cell types present in the iPSC population, including motor neurons and supporting cells.

The main objectives of this study were to determine the differential expression of miRNAs between SMA iPSCs and healthy control iPSC and to investigate their potential roles in the molecular mechanisms of SMA. By leveraging state-of-the-art molecular and computational approaches including miRNA microarray and bioinformatics analysis, this study provides novel insights into the regulatory networks controlled by dysregulated miRNAs in SMA. This will not only enhance our understanding of the disease mechanisms but also pave the way for the development of potential therapeutic interventions targeting miRNA-mediated pathways. Therefore, this information is essential for the development of miRNA-based therapies for SMA as it provides a theoretical foundation on the dynamics of gene regulation especially via miRNA activity.

## Methods

### Fibroblast-derived iPSC lines

Two fibroblast-derived iPSC lines from different SMA Type 1 patients (CS77iSMA-nxx and CS32iSMA-nxx) were purchased from Cedar Sinai Biomanufacturing Center, United States. Both the SMA human fibroblast-derived iPSCs (CS77iSMA-nxx and CS32iSMA-nxx) were karyotyped and tested routinely for mycoplasma. Information can be obtained from the links as SMA human fibroblast-derived iPSCs (CS77iSMA-nxx) (https://biomanufacturing.cedars-sinai.org/product/cs77isma-nxx/) and SMA human fibroblast-derived iPSCs (CS32iSMA-nxx) (https://biomanufacturing.cedars-sinai.org/product/cs32isma-nxx/).

A fibroblast-derived iPSC line from a clinically normal donor (NHDF-iPSCs) was kindly provided by collaborator from Universiti Tunku Abdul Rahman (UTAR), Malaysia.^22^ The CS77iSMA-nxx and CS32iSMA-nxx iPSC lines were cultured according to the manufacturer’s instructions in mTeSR1 (STEMCELL Technologies, Vancouver, Canada) on hESC-qualified Corning Matrigel coating (Corning, Corning, NY, United States). The NHDF-iPSCs were maintained in Essential 8 Flex Medium (Gibco, Waltham, MA, United States) on Vitronectin coating (Gibco, United States).^22^ Before passaging the colonies, all the differentiated colonies were carefully removed in sterile conditions. The iPSCs were split every 5 to 7 days. About 70%-90% of confluent human iPSC colonies were detached using the manual passaging method (CS77iSMA-nxx), ReLeSR (CS32iSMA-nxx) (STEMCELL Technologies, Canada), and Versene (NHDF-iPSCs) (Gibco, United States). All the cell lines were maintained under a humidified 5% CO_2_ incubation at 37 °C. Culture medium was replaced daily to maintain undifferentiated state of the iPSCs.

### RNA extraction

Total RNA from the cells was isolated using Hybrid-R (GeneAll, Korea) according to the manufacturer’s protocols. Subsequently, the concentration and purity of the isolated RNA were quantified using Nanodrop spectrophotometer (PCRmax Lambda, United Kingdom) by measuring A260/A280 value. The integrity of RNA was assessed using agarose gel electrophoresis. Further validation of RNA integrity was performed using Agilent 2100 Bioanalyzer (Agilent Technologies Inc., Santa Clara, CA, United States) to determine the RNA integrity number (RIN) and presence of small RNA. The extracted sample RIN values of ≥9.0 and the ratio miRNA ranging 1%-3% were used in this experiment. The total RNA was stored at −80 °C for further analysis.

### miRNA microarray and data analysis

In order to identify differential expression of miRNA between SMA patients (*n* = 4 for CS32iSMA-nxx and n = 3 for CS77iSMA-nxx) and control cell lines (*n* = 3 for NHDF-iPSCs), miRNA microarray analysis was carried out. All gene array data are available through Gene Expression Omnibus (GEO) accession number, GSE219262. The Agilent array was designed with 8 identical arrays per slide (8 × 60K format) (Design ID: 070156). Briefly, total 50 ng RNA was dephosphorylated, ligated with pCp-Cy3, and the labeled RNA was purified and hybridized to miRNA arrays. The array was analyzed using Agilent SureScan Microarray Scanner (G4900DA). Normalized intensities were extracted using the Agilent Feature Extraction Software and the analysis were performed using Agilent GeneSpring Analysis Software version 14.9.1. The miRNAs were filtered based on statistical significance (*P* < .05 and FC > 2). The NHDF-iPSCs replicate number two and CS32iSMA-nxx replicate number two were excluded for further analysis as these samples exhibited dissimilarity among their replicates based on the PCA plot results.

### Validation of miRNA microarray by RT-qPCR

Total RNA from the same samples as used in microarray analysis was used to validate the data from the miRNA microarrays. About 250-500 ng of the total RNA sample was reverse transcribed. The 3′ ends of the miRNAs were modified to have a poly(A) tail, and the miRNAs were reverse transcribed to cDNA using a miRNA First-Strand cDNA synthesis kit (Agilent Technologies Ins., United States), as described previously.^23^ Real-time PCR reactions were performed using a specific forward primer and a universal reverse primer in Light Cycler 480 (Roche, Basel, Switzerland).

The thermocycling conditions were as follows: initial holding stage one cycle at 95 °C for 10 min followed by 40 amplification cycles of denaturing step at 95 °C for 10 s and primer annealing at 60 °C for 15 s and extension step at 72 °C for 20 s. Primers used were hsa-miR-663a (Forward: 5′-AGGCGGGGCGCCGCGGGACCGC-3′)^24^ and U6 (Forward: 5′-CTCGCTTCGGCAGCACA-3′) (Reverse: 5′- AACGCTTCACGAATTTGCGT-3′).^25^ In each reaction, non-template control was included by substituting cDNA with water to ensure no contamination in the PCR reactions. Melting curve analysis of amplification products was performed at the end of each PCR to confirm that only one product was amplified and detected. The levels of miRNA were normalized using U6 as an endogenous control. The relative levels of hsa-miR-663a were calculated using the comparative CT (2^−ΔΔCT^) method. The RT-qPCR was performed in triplicates for NHDF-iPSCs and CS32iSMA-nxx and quadruplicates for CS77iSMA-nxx.

### Prediction of potential putative gene targeted by hsa-miR-663a

A single miRNA is known to be able to target multiple mRNAs. Thus, a computational approach using different databases to facilitate the process of narrowing down the common genes targeted by hsa-miR-663a was performed. Four different target prediction tools including TargetScan Release 7.2: March 2018 (http://www.targetscan.org/vert_72/), miRDB version 6.0 (http://www.mirdb.org/), DIANA-microT-CDS, web server version 5 (http://diana.imis.athenainnovation.gr/DianaTools/index.php?r=microT_CDS/index), and miRWalk (http://mirwalk.umm.uni.heidelberg.de/) were used in this study.

### Gene list enrichment and pathway analysis

The identified common genes were applied into ToppGene Suite (https://toppgene.cchmc.org/). ToppFun tool of the ToppGene Suite was used to discover the miRNA-gene regulatory network on the basis of biological processes and cellular compartment and disease. The threshold of significance gene-enrichment analysis was defined by *P* < .05 and Benjamini-Hochberg false discovery rate procedures were regarded as significant for the particular annotation. The *P*-value method was a hypergeometric probability mass function. The pathways relevant to the putative target genes were examined using Kyoto Encyclopedia of Genes and Genomes (KEGG) Mapper (https://www.genome.jp/kegg/tool/map_pathway1.html).

### Statistical analysis

Each experiment was carried out in triplicate at least, and the results were presented as mean ± SD using GraphPad Prism 6. Student *t*-test was used to assess statistical significance. A *P*-value of < .05 was considered significant.

## Results

### Identification of hsa-miR-663a in SMA patient fibroblast-derived iPSCs

**Figure 1.**
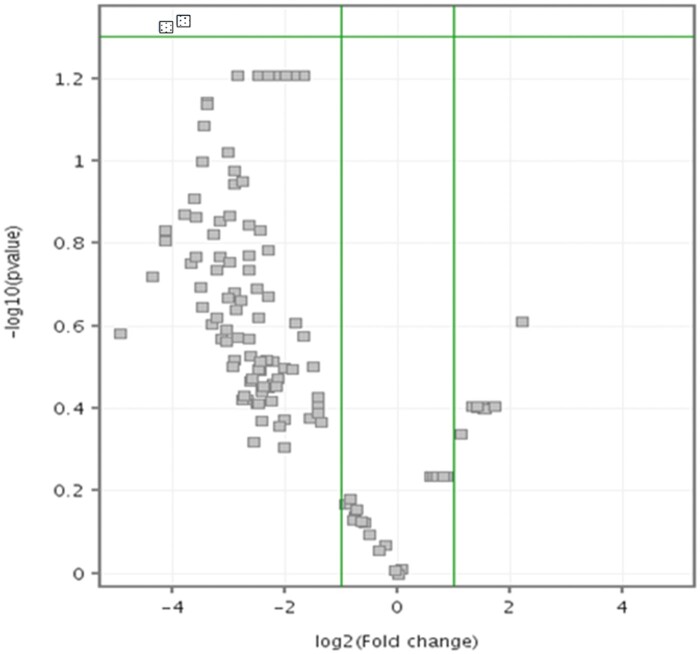
Volcano plot of divergently expressed miRNAs in the SMA patient fibroblast-derived iPSCs (CS77iSMA-nxx and CS32iSMA-nxx) relative to healthy control fibroblast-derived iPSCs (NHDF-iPSCs). Dotted square boxes represent the two downregulated miRNAs (hsa-miR-3940-5p and hsa-miR-663a). Each sample group consisted of at least three replicates.

Comparison of the expression levels of the miRNAs from miRNA microarray in SMA patient fibroblast-derived iPSCs (CS77iSMA-nxx and CS32iSMA-nxx) relative to healthy control fibroblast-derived iPSCs (NHDF-iPSCs) is depicted in Figure 1. The volcano plot graph shows two downregulated miRNAs (hsa-miR-3940-5p and hsa-miR-663a), represented as blue squares on the upper left side of the volcano plot.

The hsa-miR-663a, which was found in Chromosome 20 was selected in this study for further analysis as studies have shown the involvement of miR-663a in neuronal differentiation which affect the expression of multiple genes.^26^^,^^27^

### Validation of hsa-miR-663a in SMA patient fibroblast-derived iPSCs by RT-qPCR

**Figure 2.**
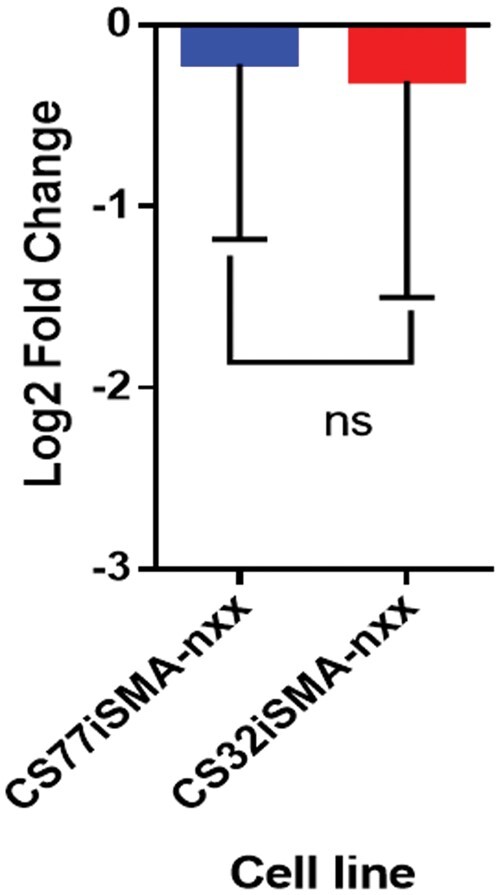
Expression of hsa-miR663a in SMA patient fibroblast-derived iPSCs (CS77iSMA-nxx and CS32iSMA-nxx) relative to control fibroblast-derived iPSCs. Error bars represent mean ± SD; ns indicates non-significant. Each sample group consisted of three replicates.

In order to validate the results from miRNA microarray analysis, the differentially expressed hsa-miR-663a between SMA fibroblast-derived iPSCs and control fibroblast-derived iPSCs was analyzed using RT-qPCR as shown in Figure 2. Results from RT-qPCR showed that hsa-miR-663a was differentially expressed in SMA patient fibroblast-derived iPSCs relative to control fibroblast-derived iPSCs. This was in concordance with the findings from miRNA microarray, which demonstrated that expression level of hsa-miR-663a was significantly lower in both SMA patient fibroblast-derived iPSCs as compared to control fibroblast-derived iPSCs.

### Predicted gene targets by hsa-miR-663a

**Figure 3.**
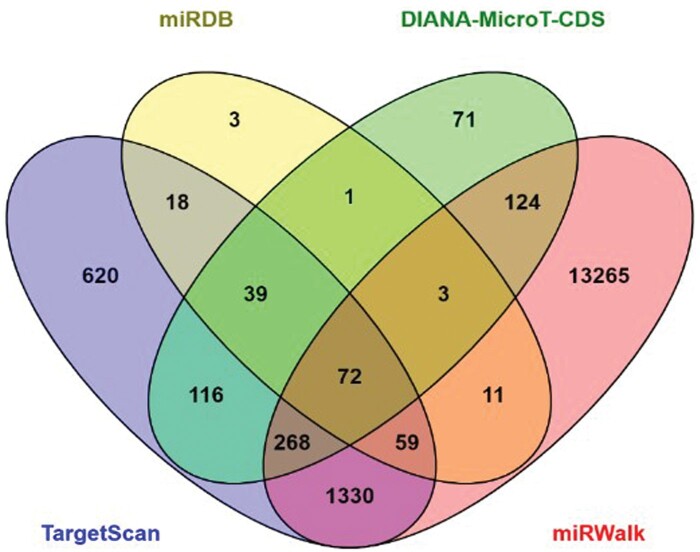
Venn diagram analysis of gene targets of hsa-miR-663a. The intersection of the four circles represents overlapping 72 gene targets among four databases including TargetScan, miRDB, DIANA-microT-CDS, and miRWalk.

The identification of miRNA target genes is essential to further explore the potential functions of hsa-miR-663a. Four databases were used to predict the common target genes. TargetScan predicted 2522 genes, miRDB 206 genes, DIANA-microT-CDS 694 genes, and miRWalk 15132 genes. The Venn diagram (Figure 3) shows the intersection of common genes targeted by hsa-miR-663a as predicted by the four databases. The list of genes from the intersection of the four databases is listed in Table 1.

**Table 1.** Identified target genes of hsa-miR-663a from 4 databases.

miRNA	Target genes
hsa-miR-663a	PCSK1N, CRTC1, PABPC1L2A, NRARP, LSP1, FGFRL1, SLC22A13, PRRT1, DDA1, NFIX, SIRT6, CMIP, STX1A, PAX2, SH3GL1, DPF1, ZNF385A, CNN2, GNG7, IGF2, MMP25, SHOX, RAB11B, ISLR2, NTN5, PPP5C, DPP9, ZBTB7A, GRIN2D, ZNF787, CYTH1, TMEM143, CARM1, NRGN, ST3GAL2, SLC25A40, FOXRED2, IGHMBP2, SEC14L2, GLO1, PHF12, SYT7, NIPAL1, TTC22, NLE1, SLC25A44, SPTBN4, DUOXA2, ADAR, ASB16, ADCYAP1R1, OLFM2, WIPF3, AKAP3, IQCE, MYO1D, KCNAB3, SH2D1B, PLA2G6, SHH, KLC2, NCDN, CACNA2D2, ZFP3, ESPN, SLC12A5, PITPNM3, SRGAP1, TNN, ZBTB7B, DBN1, GGCX.

### Functional enrichment analysis of predicted targets of hsa-miR-663a

**Figure 4.**
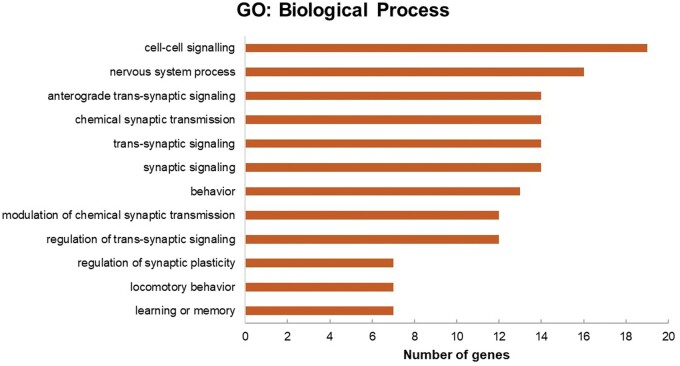
GO classification based on biological processes for 72 putative target genes of hsa-miR-663a. Gene enrichment analysis for the putative target genes predicted by four in silico prediction algorithms was performed using ToppFun.

**Figure 5.**
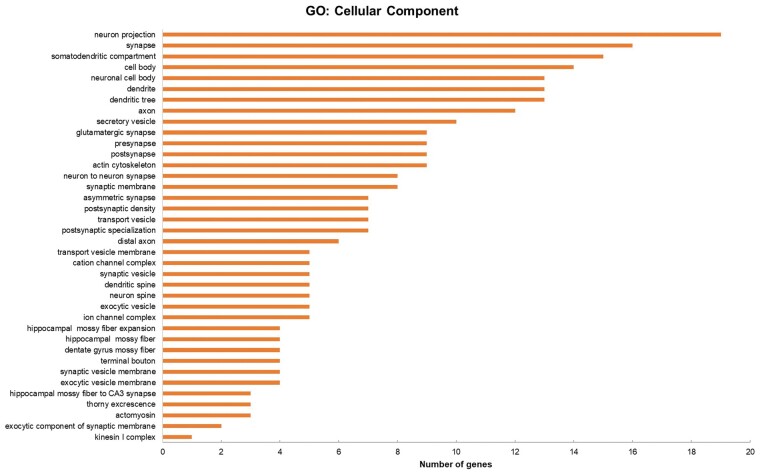
GO classification based on cellular components for 72 putative target genes of hsa-miR-663a. Gene enrichment analysis for the putative target genes predicted by four in silico prediction algorithms performed using ToppFun.

In order to explore the function of hsa-miR-663a and understand their relevant role in dysregulation of specific gene expression, the identified 72 putative genes listed in Table 1 were analyzed using ToppFun (ToppGene Suite). The functional enrichment analysis based on gene ontology (GO) from ToppFun revealed that these identified 72 putative genes are categorized to biological process and cellular components as shown in Figures 4 and 5, respectively. The GO term enrichment analyses indicated that in the biological process category, miR-663a targets were significantly enriched in the regulation of 12 categories (*P* < .05) while cellular component analysis showed that most of the target genes were mainly enriched in the neurons.

### Identification of potential pathways associated with hsa-miR-633a

**Figure 6.**
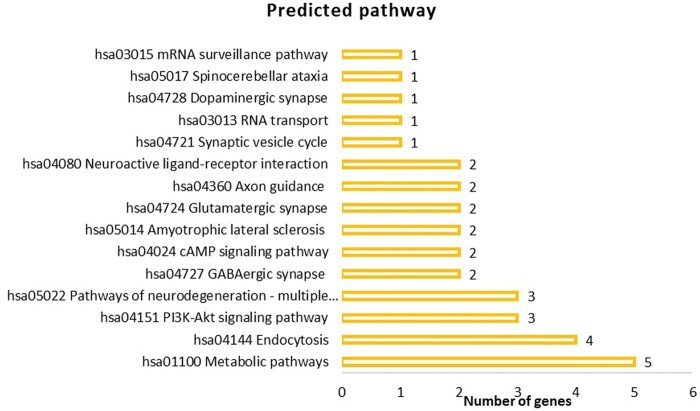
Pathway analysis was performed using KEGG mapper for 72 putative target genes of hsa-miR-663a predicted by 4 in silico prediction algorithms.

KEGG pathway analysis was performed using KEGG mapper based on the predicted target genes. KEGG pathway resulted in 95 pathways but the pathways that were predicted to be relevant to SMA is shown in Figure 6 and Table 2. Among the predicted pathways, phosphatidylinositol 3′-kinase (PI3K)-AKT signaling pathway was further explored.

**Table 2.** List of KEGG pathway relevant to SMA.

KEGG ID	KEGG pathway	Predicted target genes
hsa03015	mRNA surveillance pathway	PABPC1L2A
hsa05017	Spinocerebellar ataxia	GRIN2D
hsa04728	Dopaminergic synapse	GNG7
hsa03013	RNA transport	PABPC1L2A
hsa04721	Synaptic vesicle cycle	STX1A
hsa04080	Neuroactive ligand-receptor interaction	ADCYAP1R1, GRIN2D
hsa04360	Axon guidance	SHH, SRGAP1
hsa04724	Glutamatergic synapse	GNG7, GRIN2D
hsa05014	Amyotrophic lateral sclerosis	GRIN2D, KLC2
hsa04024	cAMP signaling pathway	ADCYAP1R1, GRIN2D
hsa04727	GABAergic synapse	GNG7, SLC12A5
hsa05022	Pathways of neurodegeneration—multiple diseases	GRIN2D, KLC2, STX1A
hsa04151	PI3K-AKT signaling pathway	GNG7, IGF2, TNN
hsa04144	Endocytosis	CYTH1, RAB11B, SH3GL1, WIPF3
hsa01100	Metabolic pathways	GGCX, GLO1, PLA2G6, SIRT6, ST3GAL2

The emphasis on the PI3K-AKT pathway was driven by earlier studies underscoring the significance of PI3K-AKT pathways in neuronal functions as highlighted in the study by Alegría and colleagues.^28^ Aberrant apoptosis is a key process that is highly likely to contribute to the pathogenesis of SMA.^29^ Thus, it is speculated that dysregulated expression of miR-663a might lead to aberrant PI3K-AKT signaling pathway triggering apoptosis in cells of SMA patients. Furthermore, *GNG7*, *IGF2*, and *TNN* are crucial for the survival and growth of motor neurons. For example, TNN is expressed highly by neurons as compared by glial cells in the central nervous system (CNS).^30^ Interestingly, another study reported that IGF2 prevented the degeneration of motor neurons derived from SMA patient iPSCs which exhibited longer neurite lengths in comparison to untreated motor neurons.^31^

### PI3K-AKT signaling pathway

**Figure 7.**
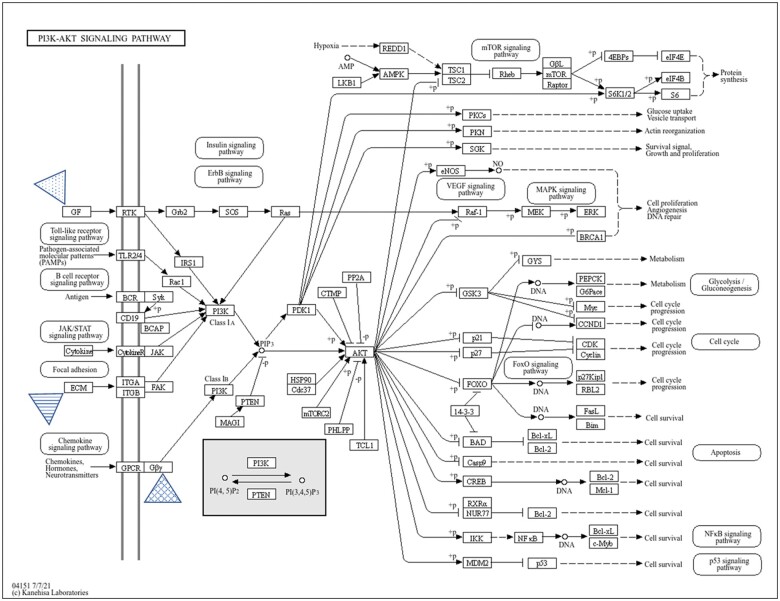
KEGG pathway analysis of phosphatidylinositol 3′-kinase (PI3K)-AKT signaling pathway with targeted genes. The involvement of genes that were associated with hsa-miR-663a; *GNG7* (diamond grid triangle arrow), *TNN* (horizontal stripe triangle arrow), and *IGF2* (dotted triangle arrow).

From the KEGG analysis, three genes (*GNG7*, *TNN*, and *IGF2*) were identified in the PI3K-AKT signaling pathway as shown in Figure 7. This pathway is known to regulate fundamental cellular functions namely transcription, translation, proliferation, growth, and survival.

## Discussion

miRNAs have been identified as a promising tool for distinguishing various diseases by modulating gene expression leading to alteration in cellular pathways and change in the cell physiology.^32^^,^^33^ It is known that miRNAs play different functions at different stages of development in spinal motor neurons.^19^ Hence, miRNAs could contribute to SMA pathogenesis in a variety of ways, including an interruption of normal miRNA biogenesis. The miRNA biogenesis is a stepwise procedure regulated by particular enzymatic complexes.^19^ It was shown that *SMN* binds directly to fragile X mental retardation protein (FMRP) and KH-type splicing regulatory protein (KSRP), which are crucial for miRNA biogenesis and function.^34^^,^^35^ Termination of proper miRNA biogenesis via deletion of Dicer in post-mitotic spinal motor neurons shows SMA-like neurodegenerative phenotype causing motor neuron dysfunction and cell death.^36^ This has clearly showed that miRNAs are essential in mature spinal motor neurons for their survival.

To date, several studies have reported the involvement of miRNAs in the pathogenesis of SMA, ie miR-9, miR-34, miR-132, miR-206, miR-183, miR-431, miR-335-5p, miR-146a, miR-375, and miR-23a.^12^^,^^19^^,^^20^^,^^37^^,^^38^ However, most of the studies were conducted in a murine model of SMA.^36^^,^^39^ A study performed by Haramati et al was the primary evidence that indicated the importance of miRNA dysregulation especially miR-9 in a genetic mouse model of SMA.^36^ Meanwhile, another study characterized the expression of 3 miRNAs such as miR-9, miR-206, and miR-132 in the spinal cord, skeletal muscle, and serum from SMA transgenic mice, and in serum from SMA patients.^40^ Their findings demonstrated differential expression of all 3 miRNAs across all the samples analyzed.

Thus, it is essential to identify the specific miRNAs that might be dysregulated to deeply understand their role in causing pathogenesis of SMA and to aid in the advancement of new therapeutic targets. Seeing that miRNAs show tissue-specific roles based on their cell- and organ-specific expression patterns,^41–43^ it is of great interest to study their differential expression levels in iPSCs derived from SMA patients. This study utilized iPSCs from SMA patients, which offer distinct advantages in terms of scalability, reproducibility, and accessibility. Moreover, utilizing of iPSCs allows for more precise control over experimental conditions and minimize potential variability arising from the differentiation process as compared to iPSC-derived neurons. Conversely, the iPSC-derived neurons may often result in heterogeneous populations of neurons with varying degrees of maturity and functionality, posing challenges in interpretation of experimental results due to confounding factors.

One of the miRNAs identified from the miRNA microarray in this study was hsa-miR-663a, which was downregulated in SMA patient fibroblast-derived iPSCs as compared to healthy control fibroblast-derived iPSCs. Previous studies reported that miR-663a is an inflammation-related miRNA that is often reported in human cancers.^25^ The dysregulation of this miRNA has been reported in colon cancer,^25^ pancreatic cancer,^44^ osteosarcoma,^45^ and non-small cell lung cancer.^46^

On the other hand, limited studies have explored the role of miR-663a in neurodegenerative diseases. A recent study revealed that the expression of miR-663a was significantly increased in the blood of patients with sporadic amyotrophic lateral sclerosis (ALS), a progressive neurodegenerative disorder that affects motor neurons, similar to SMA.^47^ This may be due to the properties of miRNAs that simultaneously target multiple and functionally related genes. Therefore, differential expression of miR-663a has been observed in different disease states.^48^

Additionally, differential expression of miR-663a was reported in TDP-43 knockdown cultured cells. TDP-43, a protein component of neuronal inclusions in neurodegenerative diseases such as frontotemporal degenerations and ALS underscoring its role in disease pathogenesis.^49^ While the role of miR-663a is still unclear, our findings suggest it might be involved in the development of other motor neuron diseases such as SMA. miR-663a should be explored further as it has great potential to be a diagnostic biomarker or a therapeutic target in SMA as its expression was found to be decreased in SMA patients as compared to the control group.

Based on the in silico prediction, *SMN1* and *SMN2* genes were not targeted by hsa-miR-663a. Thus, it is speculated hsa-miR-663a exerts an indirect effect on human *SMN* genes. A single miRNA can interact with a number of genes and a gene can be targeted by numerous miRNAs. Hence, this could result in a number of possible interactions.^50^ The differences in computational prediction analysis and experimental data could be explained by the limitation of in silico tools. These tools have different parameters which often resulted in in vast prediction outcomes with few overlaps or otherwise over-prediction resulting in large overlapping target lists.^51^ Hence, it is challenging to identify which algorithm predicts the best and most trustworthy targets. Next, each cell type in metazoans has its own set of miRNA and mRNA profiles which could be explained by various cellular transcriptomes present in both normal and disease states which are yet to be added to the predictive algorithms. Thus, the potential for false positive predicted targets is highly possible.^52^

It is important to determine the target genes of the cellular miRNA for a better understanding of their regulatory role.^53^ Based on the biological processes and cellular components from GO analysis of the targeted mRNAs by hsa-miR-663a, hsa-miR-663a is a neuron-specific miRNA that could be possibly dysregulated in the targeted cells of SMA such as motor neurons. In Figure 4, one of the common putative target genes of hsa-miR-663a is spectrin beta, non-erythrocytic 4 (SPTBN4), which has been identified in cell-cell signaling, nervous system process, synaptic signaling, trans-synaptic signaling, chemical synaptic transmission, and anterograde trans-synaptic signaling processes. It was reported that SPTBN4 disorder individuals exhibit congenital neurologic deficits including neuromuscular weakness, similar to that observed in SMA patients.^54^ Meanwhile, GO enrichment analysis results showed that the cellular component was mainly enriched in the aspects of neuron projection, synapse, somatodendritic compartment, and cell body (Figure 5). These findings could be particularly relevant to SMA, suggesting impairment of these subcellular structures and macromolecular complexes may contribute to the pathogenesis of SMA, potentially impacting motor neuron function and survival. For example, it was revealed that SMN deficiency in sensory neurons led to alterations of the synapses which connect them to motor neurons in an SMA mouse model.^55^

As indicated in Figure 7, the three main target genes of hsa-miR-663a involved in the PI3K-AKT signaling pathway are *GNG7*, *IGF2,* and *TNN*. The guanine nucleotide-binding protein 7 (*GNG7*) is a member of G protein βγ subunit (Gβγ), insulin-like growth factors (*IGF2*) is a key growth factor (GF) in this pathway, and tenascin-N (*TNN*) is an extracellular matrix (ECM) glycoprotein. GNG7 protein is part of the large G protein gamma family with GTPase activity. This protein might be related to the transmembrane signaling pathway and associated with cell contact-induced growth arrest, hence stopping uncontrolled cell growth in multicellular organisms.^56^ Further, the insulin-like growth factors (IGF1) and (IGF2) functions via the IGF1 receptor causing growth and metabolic effects through the downstream PI3K/AKT pathway. Meanwhile, the IGF2 receptor is associated with the capture and degradation of extracellular IGF2 and IGF1 throughout development as it is not a signaling receptor.^57^ IGF2 is a critical factor that is required for many aspects of cellular processes including control cell numbers, growth, differentiation, and survival.^58^ Apart from this, IGF2 is needed to activate this pathway by activating the IGF1 receptor^59^^,^^60^ and thought to play a pivotal function in neuronal survival.^61^ Tenascin-N (TNN) is a novel member of tenascin family, it is composed of cysteine-rich segments, 3.5 epidermal growth factor-like repeats, 12 fibronectin type III homologous domains, and a fibrinogen-like domain. Surprisingly, this gene is expressed highly by neurons as compared by glial cells in the CNS.^30^

These findings suggest that hsa-miR-663a may act as a crucial epigenetic regulator of the expression of these genes. This pathway is known to be affected in other neurological diseases such as Alzheimer’s disease.^62^ The results suggested that reduced expression of hsa-miR-663a might lead to dysfunction of the PI3K-AKT signaling pathway triggering apoptosis in cells of SMA patients. It is well documented that apoptosis plays a crucial role in the disease progression of SMA.^29^

In general, the cell signaling pathway is required for the functioning of the cells such as for growth, migration, apoptosis, autophagy, and metabolism.^63^ The PI3K-AKT signaling pathway is a pivotal pathway for cell survival. Indeed, PI3K-mediated signaling is an important pathway for neuronal functions and survival.^28^^,^^64^ This is in accordance with previous studies that have reported that motor neurons from both mice and humans or tissues affected in SMA show a dysregulated PI3K-AKT pathway.^65^^,^^66^ One of the studies showed that inhibition of the PI3K-Akt pathway diminished *SMN* and Gemin2 at the transcriptional level in cultured mouse and human SMA motoneurons, suggesting the role of the PI3K-Akt pathway in SMA motoneurons and how its alteration contributes to the disease pathology.^66^ A recent study also revealed that inhibition of the PI3-Akt pathway in differentiated SMA human motoneurons derived from iPSCs reduced SMN protein and mRNA levels.^67^ Collectively, the PI3K-AKT pathway is essential for the survival and maintenance of motor neurons, and thus modulation of the identified miRNA could aid in understanding the activation of the PI3K-AKT cascade.

Nevertheless, additional investigations such as qPCR and Western blot are required to verify the gene expression and protein levels of hsa-miR-663a in identifying the target molecule and the molecular components involved. Further studies should investigate the exact mechanisms of miRNA-mRNA network in SMA in a large cohort of SMA patient fibroblast-derived iPSCs. In addition to using SMA patient fibroblast-derived iPSCs, the role of the identified hsa-miR-663a could be studied in SMA patient iPSC-derived motor neurons to investigate whether similar dysregulation of *SMN1* and *SMN2* genes can be observed.

## Conclusions

Overall, the miRNA microarray analysis identified hsa-miR-663a as one of the dysregulated miRNAs expressed in SMA patient fibroblast-derived iPSCs. From the bioinformatics analysis of hsa-miR-663a, the putative target genes identified include *GNG7*, *TNN*, and *IGF2*, which are predicted to be involved in the PI3K-AKT signaling pathway. This study suggested the primary involvement of miRNAs in SMA pathogenesis and changes in miRNA expression levels could play an important role in the development of SMA. Furthermore, in silico prediction showed that hsa-miR-663a might be a neuron-specific miRNA whereby its downregulation could negatively regulate the expression of the mentioned genes affecting the signaling cascades contributing to the dysfunction as observed in SMA. In conclusion, hsa-miR-663a could be a promising future target in miRNA-based therapies for SMA.
